# VEGF-Targeted Multispectral Optoacoustic Tomography and Fluorescence Molecular Imaging in Human Carotid Atherosclerotic Plaques

**DOI:** 10.3390/diagnostics11071227

**Published:** 2021-07-07

**Authors:** Pieter J. Steinkamp, Jasper Vonk, Lydian A. Huisman, Gert-Jan Meersma, Gilles F. H. Diercks, Jan-Luuk Hillebrands, Wouter B. Nagengast, Clark J. Zeebregts, Riemer H. J. A. Slart, Hendrikus H. Boersma, Gooitzen M. van Dam

**Affiliations:** 1 Department of Surgery, University Medical Center Groningen, University of Groningen, 9700 RB Groningen, The Netherlands; p.j.steinkamp@umcg.nl (P.J.S.); l.a.huisman@student.rug.nl (L.A.H.); C.j.a.m.zeebregts@umcg.nl (C.J.Z.); 2 Department of Oral & Maxillofacial Surgery, University Medical Center Groningen, University of Groningen, 9700 RB Groningen, The Netherlands; j.vonk@umcg.nl; 3 Department of Pathology & Medical Biology, Pathology Division, University Medical Center Groningen, University of Groningen, 9700 RB Groningen, The Netherlands; g.j.meersma@umcg.nl (G.-J.M.); g.f.h.diercks@umcg.nl (G.F.H.D.); j.l.hillebrands@umcg.nl (J.-L.H.); 4 Department of Gastroenterology and Hepatology, University Medical Center Groningen, University of Groningen, 9700 RB Groningen, The Netherlands; w.b.nagengast@umcg.nl; 5 Department of Nuclear Medicine and Molecular Imaging, University Medical Center Groningen, University of Groningen, 9700 RB Groningen, The Netherlands; r.h.j.a.slart@umcg.nl (R.H.J.A.S.); h.h.boersma@umcg.nl (H.H.B.); 6 Department of Biomedical Photonic Imaging, Faculty of Science and Technology, University of Twente, 7522 ND Enschede, The Netherlands; 7 Department of Clinical Pharmacy and Pharmacology, University Medical Center Groningen, University of Groningen, 9700 RB Groningen, The Netherlands; 8 AxelaRx/TRACER BV, 9700 RB Groningen, The Netherlands

**Keywords:** carotid stenosis, atherosclerotic plaque, risk assessment, molecular imaging, photoacoustic techniques, fluorescence, pathologic angiogenesis

## Abstract

Vulnerable atherosclerotic carotid plaques are prone to rupture, resulting in ischemic strokes. In contrast to radiological imaging techniques, molecular imaging techniques have the potential to assess plaque vulnerability by visualizing diseases-specific biomarkers. A risk factor for rupture is intra-plaque neovascularization, which is characterized by overexpression of vascular endothelial growth factor-A (VEGF-A). Here, we study if administration of bevacizumab-800CW, a near-infrared tracer targeting VEGF-A, is safe and if molecular assessment of atherosclerotic carotid plaques in vivo is possible using multispectral optoacoustic tomography (MSOT). Healthy volunteers and patients with symptomatic carotid artery stenosis scheduled for carotid artery endarterectomy were imaged with MSOT. Secondly, patients were imaged two days after intravenous administration of 4.5 bevacizumab-800CW. Ex vivo fluorescence molecular imaging of the surgically removed plaque specimen was performed and correlated with histopathology. In this first-in-human MSOT and fluorescence molecular imaging study, we show that administration of 4.5 mg bevacizumab-800CW appeared to be safe in five patients and accumulated in the carotid atherosclerotic plaque. Although we could visualize the carotid bifurcation area in all subjects using MSOT, bevacizumab-800CW-resolved signal could not be detected with MSOT in the patients. Future studies should evaluate tracer safety, higher doses of bevacizumab-800CW or develop dedicated contrast agents for carotid atherosclerotic plaque assessment using MSOT.

## 1. Introduction

Carotid atherosclerotic plaque rupture is a major cause of ischemic stroke, accounting for 18–25% of all stroke events [1]. Currently, carotid endarterectomy (CEA) is recommended if the degree of extracranial internal carotid artery (ICA) stenosis is >70% in symptomatic patients to prevent a second ischemic stroke event or death [2]. However, some ischemic strokes do not correlate with stenosis severity but with plaque rupture [3].

Identification of these ‘vulnerable plaques’ by obtaining information on plaque composition could be a valuable tool to select symptomatic and eventually asymptomatic patients with increased risk for ischemic stroke that would benefit from a CEA [1] There is strong evidence that vulnerable plaques show increased levels of inflammation, characterized by a thin fibrous cap, a necrotic core and increased macrophage infiltration [4,5]. Particularly, vulnerable plaques show intra-plaque neovascularization, induced by the intra-plaque release of vascular endothelial growth factor (VEGF-A) [5,6]. Earlier, our group showed uptake of the nuclear tracer ^89^Zr-bevacizumab and the near-infrared fluorescent tracer bevacizumab-800CW, both targeting VEGF-A that linked to plaque vulnerability. Successful imaging of VEGF-A to identify vulnerable, unstable plaques has been shown through positron emission tomography [6,7].

Optoacoustic imaging is a novel non-invasive imaging modality that detects absorption-induced pressure or optoacoustic waves. As such, optoacoustic imaging enables molecular imaging at depths of several centimeters with high spatial resolution [2]. Specifically, multispectral optoacoustic tomography (MSOT) can detect multiple photoabsorbers in tissue, including both intrinsic tissue chromophores (e.g., hemoglobin, deoxyhemoglobin) and exogenous contrast agents. In contrast to ultrasound (US), optoacoustic imaging has the potential to provide biological information on plaque composition, which may improve patient stratification for CEA. To date, the feasibility of MSOT for vascular imaging, including intravascular imaging, has been shown in multiple studies [8,9,10,11,12]. Ivankovic et al. showed visualization of the carotid artery with high sensitivity and spatial resolution in vivo using optoacoustic imaging [8]. More recently, MSOT was used for carotid plaque visualization [13]. Here, we study if an intravenous injection of bevacizumab-800CW, a near-infrared fluorescent tracer that has been used in pilot studies in surgical oncology, is safe in patients undergoing CEA, a vulnerable patient population with multiple risk factors of cardiovascular disease [14,15]. We aim to determine if in vivo MSOT of bevacizumab-800CW is feasible in symptomatic patients with carotid atherosclerotic plaques.

## 2. Materials and Methods

### 2.1. Clinical Trial Design

**Figure 1:**
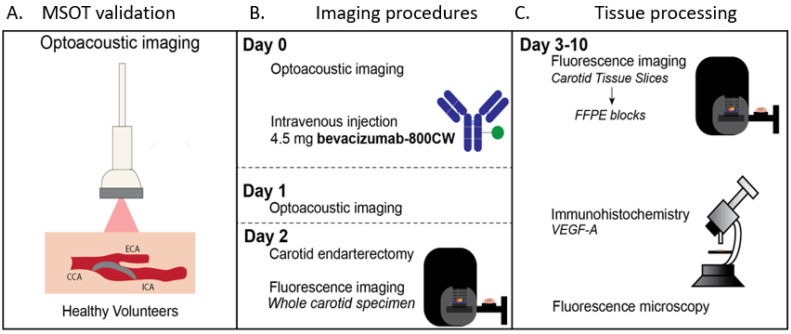
Imaging workflow. MSOT was performed on healthy volunteers for technical feasibility (**A**). On Day 0, optoacoustic imaging and tracer administration were performed, followed by bevacizumab-800CW-targeted optoacoustic imaging one day prior to carotid endarterectomy (**B**). Afterward, tissue processing using fluorescence imaging of the carotid specimen was performed (**C**). Abbreviations: MSOT, multispectral optoacoustic tomography; CCA, common carotid artery; ECA, external carotid artery; ICA, internal carotid artery.

This microdosing safety and proof-of-concept study was performed at the University Medical Center Groningen. Healthy volunteers were recruited and underwent MSOT without prior tracer administration to study technical feasibility. Subsequently, symptomatic patients (i.e., after a cerebrovascular accident or a transient ischemic attack) scheduled for CEA were included. Patients received an intravenous microdose of 4.5 mg bevacizumab-800CW three days before surgery. In this vulnerable patient cohort, an FDA-approved microdosing strategy with no direct pharmaceutical effect which gives insight in the pharmacokinetics was used to grant the safest environment for patients while still being able to study the binding of the tracer [16]. The used time interval (3 days prior to surgery) is used in a variety of oncological studies using bevacizumab-800CW based on the optimal imaging period due to the antibody half-life. MSOT was performed before and two days after tracer administration. Two carotid artery specimens from consenting symptomatic patients without tracer administration were used as a negative control. The primary endpoints were tracer safety and feasibility of bevacizumab-800CW visualization with in vivo MSOT and ex vivo fluorescence molecular imaging. The study workflow is summarized in Figure 1.

### 2.2. Safety Data

Safety for this vulnerable patient group was a primary endpoint of this clinical study, both regarding MSOT and bevacizumab-800CW administration. Bevacizumab-800CW was in-house produced in the good manufacturing practice facility of the UMCG, as previously described in detail [17]. Vital signs and physical examinations were obtained before and after MSOT and bevacizumab-800CW administration. Two weeks after tracer administration, patients were contacted by phone to identify adverse events, according to the National Cancer Institute CTCAE version 5.0 (U.S. Department of Health and Human Services, National Institutes of Health, Bethesda, MD, USA) [18].

### 2.3. Optical Characterization of Bevacizumab-800CW

Prior to the start of the clinical study, the optoacoustic profile of bevacizumab-800CW for in vivo use was studied. A tissue-mimicking phantom was fabricated using 300 mL deionized water, 2% agarose and 6% intralipid to mimic the optical properties of biological tissue. Polyethylene tubes with a diameter of 3 mm containing bevacizumab-800CW samples (diluted in deionized water) with a decreasing concentration ranging from 6.67 µM to 13 nM were inserted at a depth of ~1 cm. An IRDye800W sample with an optical density of 2 was additionally imaged as a reference. The phantom was placed in a water bath to ensure adequate coupling between the transducer and the phantom, and the samples were imaged with wavelengths from 660 to 900 nm in steps of 10 nm. Circular regions of interest ROIs of the same size were placed over the samples in the MSOT images, and subsequently duplicated at the same depth in the background to serve as a reference. The optoacoustic spectrum of bevacizumab-800CW was determined based on the highest concentration sample and compared with the IRDye800CW optoacoustic spectrum. Secondly, the maximum optoacoustic signal measured at 780 nm (i.e., optoacoustic peak) was plotted against molar concentration with background signal as a reference.

### 2.4. Optoacoustic Imaging

All MSOT procedures were performed using a clinical research hybrid ultrasound (US)—MSOT system (MSOT Acuity Echo prototype; iThera Medical GmbH, Oberschleißheim, Germany). This system uses a 25 Hz pulsed Nd: YAG laser for emission of 25 mJ pulses, which has shown clinical safety in multiple studies [19]. The two-dimensional (2D) concave detector (4-MHz center frequency, 256 transducer elements) provides cross-sectional imaging with an in-plane spatial resolution of ~180 µm and field of view of 40 × 40 mm. Simultaneously, US signal is detected for anatomical guidance of the imaging procedure.

Imaging of the common carotid artery (CCA), the carotid bifurcation and the internal and external carotid artery was performed. Subjects were in a supine position with the neck in hyperextension and the chin rotated to the contralateral side. MSOT signals were acquired using a preset with six wavelengths (700 nm, 730 nm, 760 nm, 780 nm, 800 nm and 850 nm) to sample the absorption spectrum profiles of hemoglobin (HbO2) deoxyhemoglobin (HbR) and IRdye800CW.

MSOT data were analyzed using cLabs (iThera Medical GmbH, Oberschleißheim, Germany) and reconstructed using a back-projection algorithm. The lumen of the CCA and the plaque in patient’s data were manually segmented in the US image. The lumen and plaque ROIs were duplicated at the same depth in the background region to serve as a reference. Optoacoustic signals in arbitrary units (a.u.) were extracted from the ROIs on three consecutive frames and average optoacoustic signals were registered. For image reconstruction, single wavelength analyses were performed at 800 nm to detect both HbO2 and HbR, 850 nm to detect HbO2 and 780 nm to detect IRDye800CW [20]. In addition, spectral unmixing was performed to study HbO2, HbR and IRDye800CW signal.

For quantitative analysis, the mean intensity ratios between the ROI and the background ROI were calculated to allow for data comparison of different patients, as reported earlier [13]. As such, the effects of imaging depth and light fluence are minimized.

### 2.5. Ex Vivo Analyses of Surgical Specimen

Directly after CEA, the atherosclerotic plaque was imaged using a closed-field fluorescence camera system (PEARL Trilogy, LI-COR Biosciences, Lincoln, NE, USA). Subsequently, the specimen was formalin-fixed for 24 h and serially sliced in ~1 mm thick tissue slices. Fluorescence flatbed scanning of tissue slices was performed using the Odyssey^®^ CLx (LI-COR Biosciences) and embedded in paraffin blocks (formalin-fixed paraffin-embedded [FFPE]-blocks).

ROIs were drawn of the plaque within the surgical specimen using white light images with plaque anatomy as a reference. Mean fluorescence intensities (MFI) of the bevacizumab-800CW group and the negative control from all tissue slices (2–3 tissue slices per patient) were calculated. The 4 µm tissue sections were obtained of all FFPE-blocks and hematoxylin and eosin (H&E) staining and VEGF immunohistochemistry were performed to correlate imaging results with histology. A pathologist (GFHD) blinded for imaging results analyzed all H&E tissue sections.

To assess the presence of intact bevacizumab-800CW in the atherosclerotic plaque, we performed sodium dodecyl sulfate polyacrylamide gel electrophoresis (SDS-page) on one sample of a fresh frozen surgical carotid artery specimen, as previously described. Bevacizumab-800CW was yielded in concentrations of 10 to 80 μg total protein on a biorad mini protean TGX precast gel (7.5%). Gels were stained with imperial protein stain (Thermo Scientific). Unlabeled bevacizumab was used as a reference. Subsequently, we scanned the gel using the 800 nm channel of the Odyssey CLX ^®^ flatbed scanning system (LI-COR Biosciences, Lincoln, NE, USA).

### 2.6. Statistics

Due to this proof-of-concept study’s explorative character and the subsequent small sample size, only descriptive statistics were used. MFI was defined as total counts per ROI pixel area (signal/pixel). Descriptive statistics and graph design were conducted using Graphpad Prism version 8 (Graphpad Software, San Diego, CA, USA).

## 3. Results

### 3.1. Validation of Bevacizumab-800CW Detection Using a Clinical MSOT System

**Figure 2:**
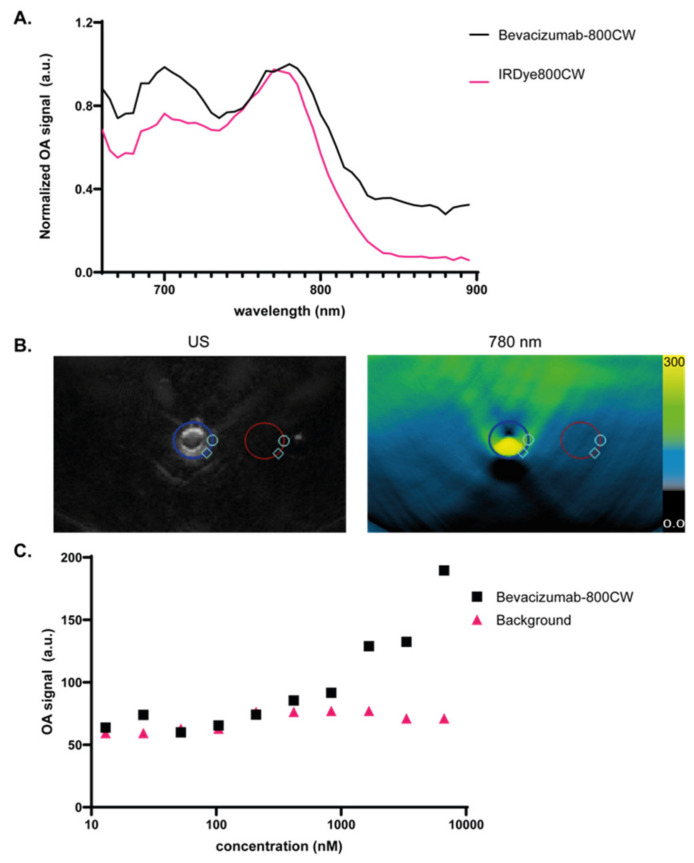
Optoacoustic characterization of bevacizumab-800CW. (**A**) Optoacoustic spectra of bevacizumab-800CW and IRDye800CW in a tissue-mimicking phantom at ~1 cm depth as determined with the MSOT Acuity Echo prototype. Two optoacoustic peaks are detected at 700 and 780 nm, with bevacizumab-800CW showing higher signal at 700 nm. (**B**) Ultrasound and MSOT 780 nm images of one of the samples imaged with corresponding ROIs of samples (blue) and background (red). (**C**) MSOT spectra of bevacizumab-800CW and IRDye800CW show two distinct peaks at 700 and 780 nm, with bevacizumab-800CW showing higher signal at 700 nm. Maximum optoacoustic signal observed with MSOT showed strong correlation with bevacizumab-800CW concentration (Pearson r = 0.96). The detection limit for bevacizumab-800CW in this setup was 416 nM.

The optoacoustic spectrum of bevacizumab-800CW and IRdye800CW were determined by imaging the different samples in a tissue-mimicking phantom at ~1 cm depth. Optoacoustic spectra were normalized to a maximum of 1. Both samples showed optoacoustic peaks at ~700 and ~780 nm, with bevacizumab-800CW showing slightly higher signal at ~700 nm, similar to previous studies with IRDye800CW conjugated to a monoclonal antibody (Figure 2). The second of IRDye800CW-peak at ~700 nm has previously been identified, and is possibly caused by stronger optoacoustic generation efficiency attributable to H-aggregates [21].

A strong correlation was found between maximum optoacoustic signal observed with MSOT and bevacizumab-800CW concentration (Pearson r = 0.96). Bevacizumab-800CW could be differentiated from the background signal down to a limit of 416 nM.

### 3.2. Subject Characteristics and Safety Data

Five healthy volunteers and five patients with symptomatic carotid artery stenosis were included in this study. Since we could not detect bevacizumab-800CW with MSOT in vivo, the trial was stopped before completing the target patient sample size (n = 10). In total, four patients (all males, mean age 76) underwent the complete in- and ex vivo imaging protocol. For one patient who received bevacizumab-800CW, the surgical plan was converted to carotid artery stenting resulting in no surgical specimen obtainment. In all patients, the plaque was localized in the internal carotid artery and CCA in all patients, and at least partially involved the ventral wall of the CCA. Patient and healthy volunteer characteristics and safety data are shown in Table 1. No drug-related or imaging related (serious) adverse events were observed.

### 3.3. In Vivo MSOT

**Figure 3:**
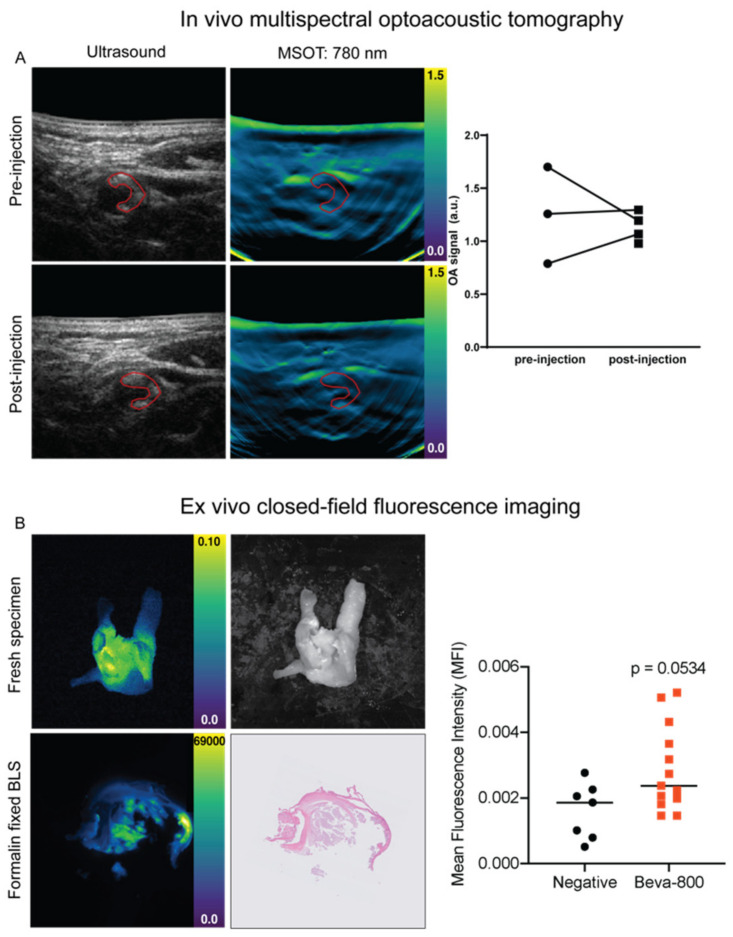
Macroscopic optoacoustic and fluorescence imaging. Representative hybrid optoacoustic image of carotid atherosclerosis pre- and post-injection (**A**), the region of interest resembling the carotid plaque used for measurements is delineated with a red line. Pre- and post-injection measurements of 780 nm optoacoustic imaging of all carotid arteries showed no difference on 780 nm and IRDye800CW. Fluorescence imaging of a whole surgical carotid specimen and a formalin-fixed tissue slice with standard histopathology (**B**). Mean fluorescence intensities (PEARL) of the plaque in all surgical tissue slices from negative patients and bevacizumab-800CW patients. Abbreviations: MSOT, multispectral optoacoustic tomography; HbR, deoxygenated hemoglobin; HbO2, oxygenated hemoglobin; HbT, total hemoglobin.

Healthy volunteer imaging showed the technical feasibility of in vivo optoacoustic visualization of the carotid artery and detection of intrinsic tissue chromophores such as HbR and HbO2 (Supplementary Figure S1). In all patients, handheld MSOT enabled real-time visualization of the CCA, internal carotid artery and external carotid artery, and identification of the atherosclerotic plaque. Although the plaque was localized in the internal carotid artery and CCA in all patients, we used only the CCA for analyses, as the light fluence in the internal carotid artery was limited by absorption of the external carotid artery. Strong absorption of hemoglobin restricted further penetration light, resulting in a “cap” of optoacoustic signal in the CCA. In all images, the optoacoustic intensity in the CCA was higher than that of surrounding tissue, both when using single wavelength analyses and spectral unmixing. (Figure 3A).

Subsequently, we assessed the ability of MSOT to identify vulnerable plaques by detecting bevacizumab-800CW signal within the atherosclerotic plaque. Pre-injection and post-injection data of single wavelength analysis at 780 nm and IRDye800CW spectral unmixing were analyzed per patient. In one patient, no pre-injection MSOT was performed due to the unavailability of the dedicated MSOT room, resulting in a total of seven data points. Upon spectral unmixing, no optoacoustic signal of bevacizumab-800CW could be detected with MSOT. In addition, no increase in single wavelength analysis of 780 nm was observed after tracer administration (1.13 (IQR 1.00–1.27) a.u. post-injection vs. 1.26 (IQR 0.79–1.17) a.u. pre-injection.

### 3.4. Ex Vivo Analyses

**Figure 4:**
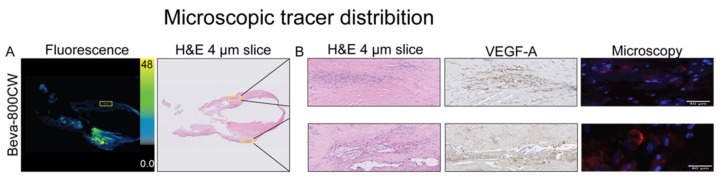
Microscopic tracer distribution. Fluorescence imaging of a formalin-fixed paraffin-embedded carotid tissue section of a vulnerable atherosclerotic plaque (**A**,**B**). Both regions with VEGF-A overexpression and calcifications due to autofluorescence show high fluorescence signal, which cannot be attributed to VEGF-specific signal (**B**). Abbreviations: VEGF, vascular endothelial growth factor A.

To further evaluate specific bevacizumab-800CW tracer uptake in the atherosclerotic plaque, we performed closed-field fluorescence imaging of the freshly excised surgical specimen. Macroscopically, fluorescence signal correlated well with areas within the plaque containing calcifications (Figure 4A). To evaluate whether the fluorescence signal was derived from the tracer, we included two carotid plaque specimen of consenting negative controls. Tissue slices of the experimental group showed an MFI of 2.37 (IQR 1.90–3.99) × 10^−4^ compared to 1.86 (0.79–2.26) × 10^−4^ in negative controls (Figure 3B).

Analysis of 10 μm sections showed increased MFI compared to the vascular wall (Figure 4A). In the atherosclerotic plaques, mainly large fibrotic and necrotic tissue was observed upon H&E histopathological examination (Figure 4A). A representative image of high VEGF-A expression in the carotid artery is demonstrated in correlation with a fluorescence signal (Figure 4B). The presence and integrity of bevacizumab-800CW within the excised vulnerable plaque was demonstrated using SDS-page on a fresh frozen excised specimen (Supplementary Figure S2).

## 4. Discussion

Currently, no imaging method can differentiate vulnerable from non-vulnerable atherosclerotic plaques with adequate accuracy for clinical use to assess for risk stratification and patient selection prior to surgery. To our knowledge, this is the first proof-of-concept study that aimed to visualize vulnerable atherosclerotic plaques by MSOT using bevacizumab-800CW for VEGF expression as a biomarker of intra-plaque neovascularization. Our data demonstrate that microdosing 4.5 mg bevacizumab-800CW is safe in a vulnerable patient population undergoing CEA. Although we could not detect bevacizumab-800CW-specific signal through in vivo MSOT, accumulation of intact bevacizumab-800CW in the carotid plaque specimen was objectified with SDS-page.

In all patients, we could visualize the entire carotid artery bifurcation area and identify the atherosclerotic plaques. Using both single wavelength analyses and spectral unmixing we could differentiate several intrinsic tissue chromophores, regardless of different imaging depths affecting light fluence. However, 4.5 mg bevacizumab-800CW could not be detected with MSOT to visualize intra-plaque neovascularization within plaques. Further ex vivo analysis with fluorescence imaging systems neither proved bevacizumab-800CW-specific signal. Nevertheless, the accumulation of bevacizumab-800CW was demonstrated through SDS-page (Supplementary Figure S2). We surmise that a microdose bevacizumab-800CW does not result in a detectable increase in optoacoustic signal, or fluorescence signal surpassing plaque autofluorescence.

The findings of this study are in line with previous studies that showed accumulation of bevacizumab in carotid atherosclerotic plaques expressing VEGF-A. Recently, our group has shown that the conjugate bevacizumab-800CW can discriminate between vulnerable and non-vulnerable plaques, and identify intraplaque angiogenesis by targeting VEGF-A [22]. Although the accumulation in atherosclerotic plaques was detected with ^89^Zr-bevacizumab micro-PET in ex vivo CEA specimens, which in fact correlated with VEGF immunohistochemistry scores, we could not detect bevacizumab-800CW in the current dose with neither MSOT or fluorescence imaging [7,14,23].

We hypothesize that the inability to detect bevacizumab-800CW with MSOT or fluorescence imaging is due to two causes. First, the current microdose might be too low to be detected or surpass the auto fluorescence in the atherosclerotic plaque. Secondly, the photophysical properties of the tracer are not optimal for optoacoustic signal generation. Therefore, we propose two strategies for future clinical studies that pursue MSOT for identification of vulnerable plaques by targeting VEGF-A, in which is used as targeting moiety since it has shown specific discrimination between vulnerable and non-vulnerable plaques.

The first and most straightforward approach could be a dose-escalation study employing an increasing dose of bevacizumab-800CW to approximate the detection limit of optoacoustic imaging, as has been carried out in multiple clinical trials in fluorescence-guided surgery and fluorescence-guided endoscopy. An alternative approach is to conjugate bevacizumab to a dedicated optoacoustic signaling compound, currently not clinically available but has gained increasing attention last decade [24,25]. Optoacoustic contrast agents have been reviewed extensively and ideally exhibit a high molar extinction coefficient in the NIR, a characteristic absorption spectrum with sharp peaks, high photostability, high photothermal conversion efficiency, low quantum yield and favorable biocompatibility [24].

Our data processing framework is a step-up towards the standardization of dual modality optoacoustic imaging together with fluorescence imaging for visualizing plaque biology and atherosclerosis. Further studies for optimizing optoacoustic contrast agents are needed to enable non-invasive transcutaneous visualization of vulnerable plaques in vivo.

## Figures and Tables

**Table 1:** Clinical characteristics of healthy volunteers and patients who underwent bevacizumab-800CW administration and finished the study.

Characteristics	5 volunteers
Age (years), mean (range)	28 (23–38)
Sex: males, n (%)	5 (100)
Weight (kg), mean (range)	82 (74–86)
Height (cm), mean (range)	186 (172–189)
Imaging time (min) mean (range)	11 (5–12)
MSOT related adverse events	0
**Characteristics**	**4 patients**
Age (years), mean (range)	76 (58–82)
Sex: males, n (%)	4 (100)
Weight (kg), mean (range)	71.5 (65–80)
Height (cm), mean (range)	172.5 (170–178)
Total infusion time (min)	1
Imaging time (min), mean (range)	9 (5–12)
Adverse events (grade 1–4)	0
MSOT related adverse events	0
Total tissue slices analyzed	43

Data are presented in numbers with percentages (%) or means with range.

## Data Availability

All data obtained during the current study are available from the corresponding author on reasonable request.

## Supplementary Materials

The following are available online at https://www.mdpi.com/article/10.3390/diagnostics11071227/s1, Figure S1: Healthy Volunteer Imaging, Figure S2: Gel electrophoresis.
