# Supplementary material for: VEGF-Targeted Multispectral Optoacoustic Tomography and Fluorescence Molecular Imaging in Human Carotid Atherosclerotic Plaques
Source: Diagnostics (Basel). 2021 Jul 7;11(7):1227. doi: 10.3390/diagnostics11071227 (PMC8305003; doi:10.3390/diagnostics11071227)
Supplement: Supplementary file 1 [file diagnostics-11-01227-s001.zip › diagnostics-1256034-supplementary.pdf]

Supplementary Materials

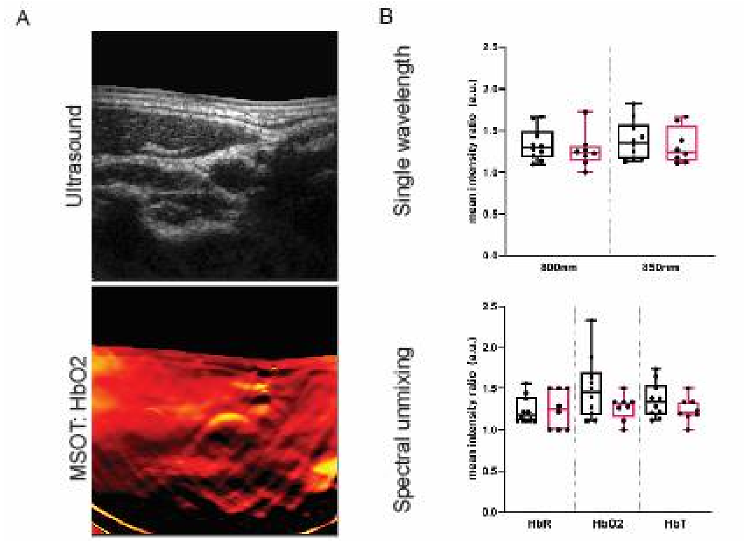

Figure S1: Healthy Volunteer Imaging. Representative image of intact bevacizumab-800CW at 150 kD. SDS-PAGE was performed on a fresh frozen tissue slice of a carotid surgical specimen collected directly after carotid endarterectomy.

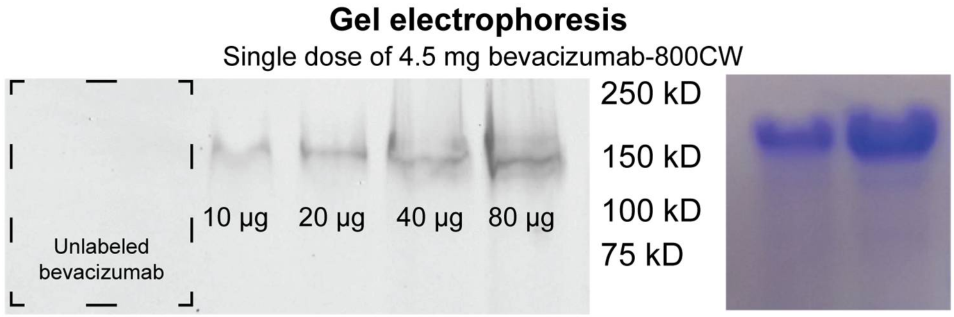

Figure S2: Gel electrophoresis. Representative image of healthy volunteer carotid artery imaging.
